# Health-related quality of life in Indonesian type 2 diabetes mellitus outpatients measured with the Bahasa version of EQ-5D

**DOI:** 10.1007/s11136-019-02105-z

**Published:** 2019-01-16

**Authors:** Bustanul Arifin, Lusiana Rusdi Idrus, Antoinette D. I. van Asselt, Fredrick Dermawan Purba, Dyah Aryani Perwitasari, Jarir At Thobari, Qi Cao, Paul F. M. Krabbe, Maarten J. Postma

**Affiliations:** 1 Unit of Pharmacotherapy, Epidemiology & Economics (PTE2), Department of Pharmacy, Faculty of Science and Engineering (FSE), University of Groningen, Groningen, The Netherlands; 2 Banggai Laut Public Hospital, Banggai Laut Local Government, Central Sulawesi, Indonesia; 3 Institute of Science in Healthy Ageing & healthcaRE (SHARE), University Medical Center Groningen (UMCG), University of Groningen, Groningen, The Netherlands; 4 Department of Health Sciences, University of Groningen, University Medical Center Groningen, Groningen, The Netherlands; 5 Department of Epidemiology, University of Groningen, University Medical Center Groningen, Groningen, The Netherlands; 6 Department of Pharmacology and Therapy, Faculty of Medicine, Public Health and Nursing, Universitas Gadjah Mada, Yogyakarta, Indonesia; 7 Bekasi General Hospital, West Java Local Government, Bekasi, Indonesia; 8 Department of Psychiatry, Medical Psychology and Psychotherapy Section, Erasmus MC University Medical Center, Rotterdam, The Netherlands; 9 Department of Developmental Psychology, Faculty of Psychology, Padjadjaran University, Jatinangor, Indonesia; 10 Faculty of Pharmacy, Ahmad Dahlan University, Yogyakarta, Indonesia; 11 Departement of Economics, Econometrics & Finance, Faculty of Economics & Business, University of Groningen, Groningen, Netherlands; 12 Department of Pharmacology and Therapy, Faculty of Medicine, Universitas Airlangga, Surabaya, Indonesia

**Keywords:** EQ-5D-5L, Index scores, Type 2 diabetes mellitus, Health-related quality of life, Indonesia

## Abstract

**Objectives:**

To present EuroQol-5D (EQ-5D) index scores in Indonesian type 2 diabetes mellitus (T2DM) outpatients and to investigate the associations between EQ-5D and socio-demographic characteristics and clinical condition.

**Methods:**

Socio-demographic data were collected by interviewing participants, clinical data were obtained from treating physicians and self-reporting. Participants originated from primary and secondary care facilities in the Java and Sulawesi regions. Ordinal regression analysis was conducted with the quintiles of the EQ-5D index scores as the dependent variable to investigate the multivariate association with the participants’ socio-demographic characteristics and clinical condition.

**Results:**

907 participants completed the five-level Indonesian version of the EQ-5D. The mean age of the participants was 59.3 (SD 9.7), and 57% were female. The overall EQ-5D index score was 0.77 (0.75–0.79). Male participants had a higher EQ-5D index score compared to females, and the highest percentage of self-reported health problems was in the pain/discomfort dimension (61%). Factors identified as being significantly associated with lower EQ-5D index scores were: (i) treatment in secondary care, (ii) lower educational level, (iii) dependency on caregivers, (iv) not undergoing T2DM therapy, and (v) being a housewife.

**Conclusion:**

This study provides estimates of EQ-5D index scores that can be used in health economic evaluations. As housewives were found to experience more T2DM-related pain/discomfort and anxiety/depression, targeted approaches to reduce these problems should be aimed specifically at this group of patients. Potential approaches could involve disease-specific-counselors (health literacy partners) who provide routine monitoring of T2DM therapy as well as improved health promotion among T2DM communities.

## Introduction

The World Health Organization (WHO) has estimated that type 2 diabetes mellitus (T2DM) will be the seventh leading cause of death in 2030 [1]. Furthermore, the U.S. Centers for Disease Control and Prevention (CDC) estimated that mortality in T2DM patients is twice as high as in people of similar age without T2DM [2]. In Indonesia, the number of T2DM patients has increased rapidly, not only in urban but also in rural areas [3], which makes Indonesia one of the countries with the most T2DM cases in the world [4]. In 2011, the International Diabetes Federation (IDF) reported that there were 7.3 million T2DM patients living in Indonesia [5] and this number had increased to 10.3 million in 2017 [4]. The Ministry of Health of the Republic of Indonesia reported, based on the comparison of T2DM data in 2007 and 2013, that new T2DM cases had doubled from 1.1 to 2.1% [3]. Recently, new cases were found in the younger age groups (15–24 years) and relatively more females than males were living with T2DM. As for the level of education, the highest percentage of T2DM was found in those who never attended school at 10.4% compared to those with a university degree at 5.9% [3]. With regard to clinical condition, 60% of T2DM patients in Indonesia have at least one T2DM-related complication, with kidney neuropathy and retinopathy being the most common complications [6, 7].

T2DM is a serious and complex chronic disease which significantly affects the daily lives of the patients, their families, and the general population in terms of premature mortality, healthcare expenditures, and lower health-related quality of life (HRQoL) [4]. Early treatment has been shown to be effective in lowering the aforementioned burdens as well as T2DM-related complications [8]. End-stage T2DM-related complications are related to higher healthcare expenditures and lower HRQoL compared to those without complications [4]. Well-structured management strategies for T2DM are warranted and interpretation and evaluation of HRQoL can help to evaluate such strategies. As the portfolio of strategies is broad and may comprise various target groups (those with advanced T2DM, those with comorbidities, those with a high dependency on a caregiver, etc.), detailed estimation for subgroups is, therefore, needed.

To our knowledge, no studies have been done to measure generic HRQoL values such as the EuroQoL-5D (EQ-5D) index scores in T2DM outpatients in Indonesia. Therefore, the aim of this study was to present generic EQ-5D index scores based on socio-demographic characteristics and clinical condition and to subsequently investigate the multivariate association between those variables. Since only the 5-level version (EQ-5D-5L) has a value set based on the Indonesian general population [9], we specifically used the EQ-5D-5L instrument in this study. We focused on two major regions in Indonesia, namely Java and Sulawesi. Java is the island where the majority of Indonesia’s population (57%) resides and can be considered representative for the western part of Indonesia [10]. Sulawesi has the highest incidence of T2DM in the whole country [3] and can represent the central and eastern part of the country.

## Methods

### Study design and setting

A cross-sectional study was carried out in Java and Sulawesi from November 2015 to October 2017 in T2DM outpatients in primary and secondary care settings. This study was approved by the Medical Ethics Committee of Universitas Gadjah Mada in Yogyakarta (KE/FK/1188/EC, 12 November 2014, amended 16 March 2015), and the Ethics Committee of Ahmad Dahlan University in Yogyakarta (011703028, 4 April 2017).

#### Java region

In the primary care setting, surveys and data collection were conducted in three family doctor clinics in Yogyakarta and a T2DM outpatient community in Surakarta (Central Java). In the secondary care setting, RSUD Dr Moewardi Hospital in Surakarta and Rumah Sehat Terpadu Dompet Dhuafa Hospital in Bogor (West Java) were chosen as the study sites.

#### Sulawesi region

Data collection was carried out at the Amirah clinic in Luwuk, Banggai (Central Sulawesi) as the study site for the primary care setting. We selected RS Akademis Jaury Hospital in Makassar (South Sulawesi) as our secondary care site.

### Participants

Patients were included in the study if they were diagnosed with T2DM by a consulting resident of internal medicine, had a minimum age of 18 years, and were willing to sign the informed consent form. For participants who were illiterate or had other difficulties with reading the form, the consent was given by the caregiver who would also further assist the participant during the subsequent data collection process.

### Instrument

The EQ-5D-5L is a generic HRQoL instrument. The paper and pencil version used in this study consists of two pages [11]. The first page is the EQ-5D classification consisting of a descriptive system that comprises five dimensions: mobility, self-care, usual activities, pain/discomfort, and anxiety/depression. Each dimension has five levels: no problems, slight problems, moderate problems, severe problems, and unable/extreme problems which represent the severity of problems for the specific dimension. A single digit expresses the level selected for that specific dimension. Therefore, the five-digit number for five dimensions describes a specific health state. For example, ‘11111’ indicates ‘no problems in any of the five dimensions,’ while ‘21134’ indicates slight problems in the mobility dimension, no problems in the self-care and usual activities dimensions, moderate problems in the pain/discomfort dimension, and severe problems in the anxiety/depression dimension. Each EQ-5D health state is then calculated to a single index score based on the preference of the relevant general population; i.e., the Indonesian value set in this case [9, 12]. For instance, the health state of ‘11111’ corresponds to the maximum EQ-5D index score of 1.00, and ‘21134’ leads to a score of 0.56. The second page of the instrument comprises the visual analogue scale, labelled EQ-VAS. This thermometer-like scale (ranging from 0 to 100) reflects the patient’s health in general, representing a more integral measure than the EQ-5D index scores [12]. In addition, the EQ-VAS represents the patient perspective, whereas the EQ-5D index score, since it uses population preferences, reflects the societal perspective. The participants were asked to rate their own health, where zero indicates the worst imaginable health state, and 100 indicates the best imaginable health state.

### Data collection procedure and data sources

To ensure a smooth process of distributing the EQ-5D-5L instrument to the participants, the researcher asked the general practitioners (GPs) and consulting residents of internal medicine who were responsible for the participants to assist by providing information about ethics, the objectives of the research and the importance of participating. Notably, it was hypothesized that participants would be more cooperative in completing the instrument when it was introduced by the treating physician. The process of distributing the instrument took place in the outpatients’ waiting rooms in the primary and secondary care settings concerned. In addition, some instruments were distributed when the participants joined the morning exercise in the T2DM community. During the data collection process, the researcher provided the material to all participants, explained the material to them, we gave them opportunity to ask related questions and assisted with filling out if needed (for example, as they had forgotten their glasses). It was not an interview but instead the participants needed further explanation on how to fill out the EQ-5D instrument.

Socio-demographic data such as gender, age, T2DM duration, occupation, level of education, and dependence on a caregiver were obtained from self-reporting. We classified the participants into two age categories based on the retirement age of Indonesian people (56 years): productive age (below 56 years) and retirement age (56 years and above). As for employment status, participants were defined as unemployed if they reported not having a job, and in active employment when they were still actively working. Those whose main responsibilities were for their family members and household chores were classified as housewives.

Data on the clinical condition such as the type of therapy, T2DM-related complications, and comorbidities were obtained from the GPs or consulting residents of internal medicine. Self-reported data from participants was used in the cases data could not be collected through GPs or residents of internal medicine. In this study, participants were defined as having comorbidities if they suffered comorbidities such as cancer, tuberculosis, gastritis, hepatitis, low back pain, urinary tract infections, and tumors, reflecting the most-reported comorbidities in the medical records of selected participants. In addition, participants with comorbidities and T2DM-related complications were considered as a separate group to be analyzed specifically.

### Statistical analysis

EQ-5D index scores were calculated using the Indonesian value set [9]. Descriptive statistics were computed to compare EQ-5D index scores among different subgroups based on socio-demographic characteristics and clinical condition; both means and 95% confidence intervals (CIs) were calculated. Univariate associations between the EQ-5D index score and various participants’ characteristics were subsequently tested by Chi square tests. Next, a multivariate ordinal regression analysis was conducted to explore how this score was associated with the socio-demographic characteristics and the clinical condition. This method was chosen because the EQ-5D index scores were too skewed to include as the dependent variable in the linear regression model. In addition, multinomial regression was not chosen as it cannot be conducted when there are no pre-defined cut-off values to differentiate the EQ-5D index scores into different subgroups. As a dependent variable in our ordinal regression model we used the quintiles of the EQ-5D index scores. We considered all variables that were separately included in the univariate analysis as being the independent variables in our multivariate analysis. The association was then investigated without deleting the insignificant independent variables. Unlike being categorical variables in the univariate analysis, the T2DM duration, fasting blood glucose (FBG), and postprandial blood glucose were entered into the model as continuous variables after comparing the corresponding goodness-of-fit of the regression models. The existence of multicollinearity in our regression model was assessed by the variance inflation factor (a value > 10 indicates multicollinearity). Missing values on T2DM duration, FBG, and postprandial blood glucose were dealt with using multiple imputations [13]. For the multiple imputation we assumed the missing values were missing at random (MAR) and we used the multivariate imputation by chained equations (MICE) technique (also called sequential regression multiple imputation) to impute the data [14]. For simplicity and to ensure more straightforward analysis, we used only continuous variables that were included in the model (i.e., not categorical variables) to predict missing values. This means that the completed dataset of T2DM duration, FBG, and postprandial blood glucose were predicted based on the observed values of these three variables. Considering the percentage of missing measurements (about 35% for both FBG and postprandial blood glucose level), 25 imputed datasets were obtained for each measurement. The completed measures were then computed by taking the average values generated from each imputed dataset. When setting up the regression, the independent variables ‘gender’ and ‘T2DM duration’ were found to not meet the proportional odds assumption [15] when using the quintile EQ-5D index score. To relax this assumption in the regression model, the effects of these two variables were allowed to be varied across the intervals of the index score (quintile 1 and 2, quintile 2 and 3, quintile 3 and 4, and quintile 4 and 5). The descriptive statistics with the corresponding tests were performed using IBM SPSS Statistics for Windows, version 25 (SPSS Inc., Cambridge, MA). The ordinal regression model was built using R (R Foundation for Statistical Computing, software version 3.4.0, Vienna, Austria). A statistically significant association was defined as having a two-tailed *p* value of < 0.05.

## Results

### Characteristics of the participants

The socio-demographic characteristics and clinical condition of the participants are shown in Table 1. In total, there were 907 participants (mean age 59.3 (SD 9.7) years) included in our study, 57% were female and about 69% of female participants reported that they were housewives. Of the 359 housewives, 60% were 56 years or older and 4% had a university degree. Almost 55% of male participants were still actively working, either for the government, a company or self-employed. In this study, almost 80% of participants had a lower educational level and 66% of participants had already retired. More than 50% of the participants were accompanied by a caregiver and the majority of caregivers were comprised of spouses or children. We adjusted the socio-demographic characteristics data that were self-reported with the participants’ identity cards if needed.

**Table 1:** Distribution of participants within different subgroups over reporting problems (slight to extreme problems) on the EQ-5D dimensions and mean (95% CI) EQ-5D index score according to socio-demographic characteristics and clinical condition in Indonesian T2DM outpatients using the Indonesian EQ-5D tariff

Characteristics	*N* (%)	% reporting problems					EQ-5D index score (95% CI)
		Mobility	Self-care	Usual activities	Pain/discomfort	Anxiety/depression	
Total participants	907 (100)	37	12	23	61	34	0.77 (0.75–0.79)
Socio-demographic characteristics							
Region							
Java	499 (55)	38	13	23	57	36	0.78 (0.75–0.80)
Sulawesi	408 (45)	37	12	23	65	33	0.76 (0.73–0.79)
Sex							
Male	387 (43)	34	11	22	58	37	0.79 (0.75–0.81)*
Female	520 (57)	40	14	24	63	37	0.76 (0.73–0.78)
Age (59.32 ± 9.70)	886 (98)^#^						
Productive age (< 56 years)	289 (32)	32*	12	25	61	43**	0.77 (0.73–0.80)
Retirement age (≥ 56 years)	597 (66)	40	12	22	60	30	0.77 (0.75–0.80)
Occupation							
Active employment	314 (35)	30**	8**	19**	59*	33**	0.81 (0.78–0.84)**
Unemployed	234 (26)	36	11	19	56	27	0.79 (0.75–0.82)
Housewife	359 (39)	45	17	30	66	41	0.72 (0.69 − 0.075)
Education							
Up to senior high school	698 (77)	41**	15**	27**	65**	37**	0.74 (0.72–0.76)**
University degree	209 (23)	24	4	12	48	27	0.86 (0.83–0.89)
Level of health facilities							
Primary care	133 (15)	20**	3**	8**	40**	41	0.90 (0.87–0.92)**
Secondary care	774 (85)	40	14	26	64	33	0.74 (0.73–0.77)
Dependency on a caregiver							
Yes	488 (54)	44**	15**	27**	64*	37	0.72 (0.69–0.75)**
No	419 (46)	29	9	19	57	32	0.83 (0.81–0.85)
Clinical condition							
T2DM duration^a^	805 (89)^#^						
< 5 years	446 (49)	38	15	29**	63	36	0.76 (0.74–0.79)
≥ 5 years	359 (40)	43	12	20	64	32	0.74 (0.71–0.77)
Therapy							
None (diet, herbal or exercise)^b^	49 (5)	47**	27**	41**	59*	45	0.61 (0.47–0.76)**
OAD (mono and combinations)	490 (55)	32	10	19	57	32	0.81 (0.79–0.83)
Insulin (mono and combination with OAD)	368 (40)	44	14	27	66	37	0.74 (0.71–0.77)
Types of complications and comorbidities							
Complications							
None	269 (30)	33	8**	14**	57	33	0.80 (0.76–0.83)*
Macrovascular	290 (32)	38	11	23	59	33	0.79 (0.72–0.82)
Microvascular	140 (15)	36	11	21	64	29	0.77 (0.72–0.82)
Macro and microvascular	30 (3)	47	23	44	56	34	0.69 (0.56–0.80)
Comorbidities^c^	86 (10)	36	20	36	69	44	0.71 (0.65–0.78)
Comorbidities + T2DM complications	92 (10)	49	22	36	67	42	0.70 (0.63–0.76)
Number of T2DM complications							
None	269 (30)	33*	8*	14**	57	33	0.80 (0.76–0.83)
1 T2DM complication	341 (37)	35	11	22	61	31	0.79 (0.76–0.81)
2 or more T2DM complications	119 (13)	48	12	29	58	37	0.74 (0.69–0.80)
Blood glucose level							
Random blood glucose	147 (16^)#^						
≤ 200 mg/dl	73 (8)	23	9	20	37	22	0.70 (0.62–0.76)
> 200 mg/dl	74 (8)	26	12	23	36	27	0.63 (0.54–0.71)
Fasting blood glucose	685 (76)^#^						
≤ 126 mg/dl	265 (30)	15	4	8	23	10*	0.79 (0.75–0.82)
> 126 mg/dl	420 (46)	24	9	15	40	21	0.75 (0.72–0.78)
Postprandial blood glucose	570 (63)^#^						
≤ 200 mg/dl	309 (34)	18*	4*	10*	32	15	0.81 (0.78–0.84)*
> 200 mg/dl	261 (29)	19	6	12	29	15	0.76 (0.73–0.79)

^#^Reporting rate: *p* value: Chi square; *p value < 0.05; **p value < 0.01

^a^11% of respondents did not know the duration of their T2DM

^b^Five participants reported that the reason for not taking metformin was the occurrence of side effects such as dizziness and nausea. Besides that, five participants with normal blood sugar levels but abnormal blood pressure levels requested that they only be given antihypertensive medication because they felt scared if they had to take more than three pills at a time

^c^Comorbidities were defined as diseases other than T2DM complications, such as cancer, tuberculosis, gastritis, low back pain, urinary tract infections, and tumors

With regard to clinical condition, almost 50% of participants had been diagnosed with T2DM in the last five years and nearly 60% were on oral anti-diabetic (OAD) therapy. In addition, 40% of participants used insulin therapy and this was relatively more prevalent among participants in secondary care settings. In this study, 30% of participants did not report any complications and 10% of participants reported comorbidities. Notably, data on clinical condition of all participants in the secondary care setting were derived from consulting residents of internal medicine (*n* = 774, 85%). For the participants in primary care the information on clinical condition was partly obtained via the GPs (*n* = 100, 11% of the total population) and the remainder (*n* = 33, 4%) was through self-reporting.

### EQ-5D dimensions affected by T2DM

In total, 61% of participants reported problems (i.e. level 2, slight problems, to level 5, unable/extreme problems) with regard to pain/discomfort and this was found to be the highest proportion among all five dimensions (Table 1). The housewives (compared to the actively employed and unemployed) and those with lower education (compared to university degree participants) reported a higher percentage of the presence of problems on all dimensions. Participants treated in secondary care (compared to those who were treated in primary care) and participants accompanied by a caregiver (compared to participants who came alone) reported a higher percentage of problems on all dimensions except for anxiety/depression. Retired participants reported a higher percentage of problems in mobility than those who were still productive, but this was the other way around for the anxiety/depression dimension. Participants on the island of Sulawesi reported a higher percentage of problems in pain/discomfort than those who lived on Java, but no significant differences were found in the other four dimensions.

With regard to clinical condition, the majority of participants on insulin therapy reported problems on the pain/discomfort dimension. In addition, participants with macrovascular and microvascular complications and those with T2DM-related complications and comorbidities reported experiencing problems on the self-care and usual activity dimensions. Moreover, a higher number of T2DM-related complications seemed to be associated with more problems on the mobility and usual activities dimensions. Of the 570 participants who had a post-prandial blood glucose test, participants with blood glucose of > 200 mg/dl also reported problems on the mobility, self-care, and usual activity dimensions.

### Univariate association between EQ-5D index scores and the participant characteristics

**Fig. 1:**
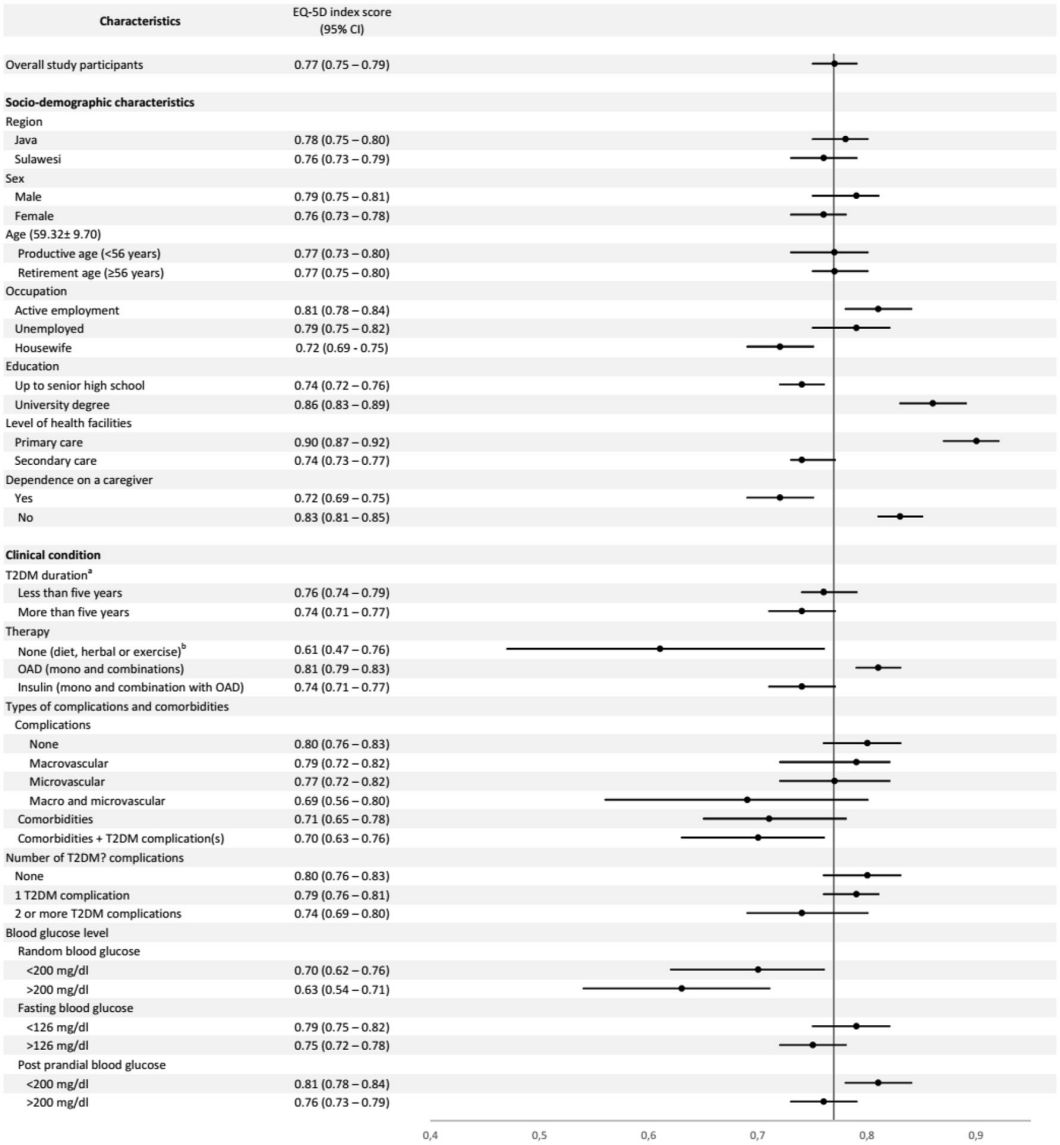
Mean (95% CI) EQ-5D index score according to socio-demographic characteristics and clinical condition in Indonesian T2DM outpatients using the Indonesian EQ-5D-5L tariff

The average EQ-5D index score in Indonesian T2DM outpatients was 0.77 (95% CI 0.75–0.79). The score of 0.77 was derived from the calculation of total EQ-5D index score of the study respondents with the Indonesian general population TTO value set [9]. Male participants had a higher EQ-5D index score compared to female participants (Table 1). Based on occupation, housewives had the lowest EQ-5D index score compared to actively employed and unemployed participants. Participants treated in secondary care and those with a lower level of education had a lower EQ-5D index score compared to those in primary care and with higher education, respectively. Furthermore, we also found that participants who were accompanied by a caregiver during a visit to a health facility indicated lower EQ-5D index scores compared with participants who came alone (Fig. 1).

With regard to clinical condition, the EQ-5D index scores in participants with OADs (mono or combination therapy) were higher than those on insulin therapy or those not undergoing therapy. Furthermore, participants with T2DM-related complications or comorbidities reported lower EQ-5D index scores than those without complications or comorbidities. In addition, participants with controlled blood sugar reported a higher EQ-5D index score compared to those who had uncontrolled blood sugar.

### Multivariate association between EQ-5D index scores and the participant characteristics

Table 2 presents the results of the multivariate ordinal regression model. No multicollinearity was detected in the model. Several characteristics of the participants were shown to significantly influence the EQ-5D index score, mostly in line with the results of the univariate analysis presented above. Participants in secondary care had a lower EQ-5D index score compared to those in primary care. Again, higher education contributed to a significantly better HRQoL for the participants in our study. A caregiver accompanying the participant was shown to be negatively associated with HRQoL. In addition, housewives had a lower EQ-5D index score compared to active employees. The variables with regard to clinical condition were all shown to not significantly influence the index score except for treatment using monotherapy and combinations of OADs. Not surprisingly, participants having treatment using OADs had a two-fold EQ-5D index score compared to those who were not being treated using OADs.

**Table 2:** Association between socio-demographic characteristics, clinical condition and EQ-5D index scores using a multivariate ordinal regression model

Variables	Coefficient (95% CI)	*p* value
Socio-demographic characteristics		
From Sulawesi (vs from Java)	0.88 (0.68–1.14)	0.344
Secondary care (vs primary care)	0.32 (0.23–0.48)	< 0.001
Age	0.79 (0.60–1.05)	0.113
University degree (vs high school)	1.83 (1.33–2.53)	< 0.001
With caregiver (vs no caregiver)	0.65 (0.51–0.84)	< 0.001
Retired (vs active employee)	0.86 (0.61–1.21)	0.382
Housewife (vs active employee)	0.62 (0.42–0.91)	0.014
Female (vs male)	0.96 (0.62–1.50) [quintile 1 and 2]	0.886
	0.96 (0.66–1.42) [quintile 2 and 3]	0.858
	1.44 (0.98–2.11) [quintile 3 to 5]	0.064
Clinical condition (vs no complications/comorbidities)		
One complication	1.09 (0.80–1.48)	0.585
Two or more complications	0.74 (0.49–1.11)	0.142
Comorbidities	0.73 (0.46–1.17)	0.196
Complications and comorbidities	0.71 (0.45–1.12)	0.137
Oral antidiabetic (vs none)	2.18 (1.22–3.89)	0.008
Insulin (vs none)	1.55 (0.86–2.82)	0.147
Fasting blood glucose^a^ (*n* = 685)^b^	0.99 (0.99–1.00)	0.397
Postprandial blood glucose^a^ (*n* = 570)^b^	0.99 (0.99–1.00)	0.074
T2DM duration	1.00 (0.98–1.03) [quintile 1 and 2]	730
	0.99 (0.97–1.01) [quintile 2 and 3]	0.505
	0.98 (0.96–1.01) [quintile 3 to 5]	0.132

EQ-5D index score: 1st quintile (− 0.87 to 0.62), 2nd quintile (0.62–0.80), 3rd quintile (0.80–0.91), 4th quintile (0.91–1), 5th quintile 1

^a^Continuous variable in the multivariate model

^b^Reporting rate

## Discussion

This is the first population-based study that reported EQ-5D index scores based on socio-demographic characteristics and clinical condition in Indonesian T2DM outpatients. We found five factors that were significantly associated with lower EQ-5D index scores in our multivariate model: treatment in secondary care, lower educational level, dependency on caregivers, occupation as a housewife and not undergoing T2DM therapy. The mean EQ-5D index score in Indonesian T2DM outpatients in this study was estimated at 0.77 (95% CI 0.75–0.79).

The mean EQ-5D index score in Indonesian T2DM outpatients in our study, 0.77, is lower than that reported for the Indonesian general population at 0.91 [9]. Furthermore, a previous study by Perwitasari et al. in Indonesian T2DM outpatients (*n* = 86) reported that the EQ-5D index score was 0.75 (SD 0.22) [16]. Related to clinical condition, Perwitasari et al. also found that T2DM complications were indeed aligned with decreases in EQ-5D index scores. Notably, despite the similarities, Perwitasari et al. used different methods to measure and analyze the data, i.e., the EQ-5D-3L instrument and the Thailand TTO value set, which complicates comparison [16]. Our estimate is in line with what was previously found in a meta-analysis on EQ-5D in mostly T2DM patients at 0.76 (95% CI 0.75–0.77) [17], despite that the meta-analysis comprised populations from various backgrounds, including high, middle and low-income countries as well as various stages of disease in DM, hampering a straightforward comparison with our study.

Concerning self-reported health, the percentage of T2DM outpatients in our study that reported any problems is higher than reported for the general population in four out of five dimensions: mobility (37% and 8%, respectively), self-care (12% and 2%), usual activities (23% and 11%), and pain/discomfort (61% and 40%) [9]. Notably, for the anxiety/depression dimension, we found 34% compared to 35% in the general population [9]. This shows that T2DM has an adverse impact on HRQoL. Our finding that the EQ-5D index score in female participants is lower than in males seems to be consistent with previous studies in similar participants [18–21]. A possible explanation for this might be that female participants are more likely to report anxiety/depression problems as they have been reported to have more diabetes-related worries, less satisfied with treatment regimens, and less ability to cope with their disease [22, 23]. However, when we controlled for socio-demographic characteristics and clinical condition in the multivariate ordinal regression model, the difference between males and females was no longer significant. This may be due to the fact that 69% of the females were housewives (with 96% with lower education) and being a housewife was already significantly associated with a lower EQ-5D index score. It could be argued that for Indonesian housewives who have the responsibility for taking care of the family members and household chores, having a chronic illness such as T2DM represents an extra burden in fulfilling these tasks. Percentages in this subgroup reporting problems on all of the EQ-5D dimensions indeed confirmed this being significantly higher than in the other subgroups of actively employed and unemployed.

Some publications on HRQoL in Indonesian T2DM outpatients exist, mainly conducted on the island of Java [16, 24]. In this study, we focused on socio-demographic characteristics and clinical condition of Indonesian T2DM outpatients in two major islands, namely Java and Sulawesi. To get a better description on the characteristics of T2DM participants, we compared ours with two previous studies [16, 24]. These two studies were of particular interest as one used a previous version of the same instrument, i.e., EQ-5D [16] and the other focused on socio-demographic characteristics and HRQoL in Indonesian T2DM outpatients using the diabetes quality of life clinical trial questionnaire (DQLCTQ) (*n* = 83) [24]. Both of these studies reported that the percentage of female participants was higher than male participants. In addition to that, most participants in both studies were aged 55 years or older. Notably, some differences between our study and the other two studies concerned the percentages of participants with a university background as compared to only a high school background. A detailed description of this issue can be found in the “Appendix”.

Our findings showed that higher educational levels lead to higher HRQoL, which was similar to findings from studies in other countries, such as in Korea, Japan and Iran [18–20]. It could be argued that participants with a higher level of education might have a better understanding of the T2DM therapy and the impact of T2DM-related complications, and therefore, have a more conscientious attitude towards their therapy [21].

Participants who were treated in secondary care were found to have lower HRQoL than those who were treated in primary care. This seems reasonable since worse cases are generally referred from the primary to secondary healthcare facilities with higher severity of T2DM. Similar explanations could be given for findings on lower index scores of participants needing help from their caregivers compared to those who did not need such help: a worse condition likely involves more need for help as well as being associated with a lower HRQoL.

During the data collection process, approximately 550 participants were assisted with filling out the EQ-5D instrument, amongst which approximately 3/4 s were elderly and the rest were participants in the productive age with an education level only up to senior high school. The researcher or research assistant sat next to them, explaining and/or assisting them to read and comprehend each item. Some reasons for this assistance were that they forgot to bring their glasses, were too tired because of the bureaucracy in the hospital, or needed explanation concerning the difference between levels of the EQ-5D instrument [25, 26]. We examined whether our assistance filling out the instrument influenced our results. For that purpose, we calculated the frequencies of problems reported based on those participants who were assisted versus those not assisted and the results were similar (data not shown).

Some limitations of this study have to be acknowledged. First, we collected data only on two major islands of Indonesia, namely Java and Sulawesi. Representativeness of the study sample for the whole of Indonesia can obviously not be straightforwardly claimed. Yet, given our choice for the most densely populated central island (Java) and a more remote area (Sulawesi), we did include a spectrum in our sample covering some ethnic variety and some representativeness may definitely exist. Second, there were 21 participants that had missing information on their date of birth for privacy reasons. As these 21 participants only constitute a minor part (2%) of the total sample, and also because age was not found to be associated with EQ-5D index scores it is unlikely this has had a profound influence on our results. Lastly, the multiple imputation approach we conducted only used the evidence from the available continuous variables to predict the missing information. This did not take into account the potential influence of the categorical variables that were included in the multivariable model. We would, however, not expect this limitation to relevantly impact our results. In addition, a “rule of thumb” to use a number of imputations equal to the percentage of missing data was used as proposed by White et al. [27]. This would imply 35 imputed datasets instead of 25 in this study. However, increasing the number of imputed datasets had hardly any impact on the results presented in this study after performing a sensitivity analysis (results not shown).

## Conclusion

This study provides estimates of EQ-5D index scores that can be used in health economic evaluations. Five factors were found in our multivariate model to be significantly associated with lower EQ-5D index scores: treatment in secondary care, lower educational level, dependency on caregivers, occupation as a housewife and not undergoing T2DM therapy. Housewives are prone to experiencing T2DM-related pain/discomfort and anxiety/depression, therefore, specific approaches to reduce these problems should be aimed specifically to this group of patients. Potential approaches could involve disease-specific counselors (health literacy partners) who provide routine monitoring of T2DM therapy as well as improved health promotion among T2DM communities.

## Recommendation

We recommend the development of a specific approach targeting housewives living with T2DM and T2DM patients with lower levels of education. Given their decrease in HRQoL compared to the average T2DM outpatients in this study, there is an urgent need for improvement. Such health promotions could be integrated with existing health programmes, such as Prolanis BPJS/Badan Penyelenggara Jaminan Sosial, a targeted diabetes program run by the social security administrative agency.

## Appendix: Comparing key demographic characteristics of our study participants with two previously published articles [16, 24]

**Table:**

Characteristics	*N* (%)		
	Arifin et al. (The present study)	Perwitasari et al. [16]	Restinia et al. [24]
HRQoL instrument	EQ-5D-5L (Indonesia value set)	EQ-5D-3L (Thailand value set)	DQLCTQ
EQ-5D index score (95% CI)	0.77 (95% CI 0.75–0.79)	0.75 (SD 0.22)	NA
Overall study participants	907 (100)	86 (100)	83 (100)
Socio-demographic characteristics			
Number of study sites	8	1	1
Location	Java and Sulawesi	Singkawang, West Kalimantan	Jakarta
Sex			
Male	387 (43)	37 (43)	31 (37)
Female	520 (57)	49 (57)	52 (63)
Mean age	59.3 (SD 9.7)	58.2 (SD 7.8)	> 50 years
Age	886 (98)	86 (100)	83 (100)
Productive age (< 56 years)	289 (32)	NR	3 (4)
Retirement age (≥ 56 years)	597 (66)	NR	80 (96)
Occupation			
Active employment	314 (35)	53 (62)	3 (4)
Unemployed	234 (26)	33 (38)	80 (96)
Housewife	359 (39)	NR	NR
Education			
Up to senior high school	698 (77)	31 (36)	35 (42)
University degree	209 (23)	55 (64)	48 (58)
Level of health facilities			
Primary care	133 (15)	0 (0)	0 (0)
Secondary care	774 (85)	86 (100)	83 (100)
Dependence on a caregiver			
Yes	488 (54)	NR	NR
No	419 (46)	NR	NR
Monthly income (1 USD$ = IDR 14.835)	NR	< 2.2 million ($148): 30 (35)	< 3 million ($202): 47 (57)
	NR	>2.2 million: 56 (65)	> 3 million: 36 (43)
Family history of T2DM (yes)	NR	53 (62)	NR
Marital status (married)	NR	76 (88)	NR
Clinical condition			
T2DM duration^a^	805 (89)	Mean 6.7 (SD 4.9)	NR
Less than 5 years	446 (49)	NR	14 (17)
More than 5 years	359 (40)	NR	69 (83)
Therapy			
None (diet, herbal or exercise)	49 (5)	0 (0)	NR
OAD (mono and combinations)	490 (55)	78 (91)	NR
Insulin (mono and combination with OAD)	368 (40)	8 (9)	NR
			NR
Types of complications and comorbidities			
Complications			
None	269 (30)	0 (0)	NR
Macrovascular	290 (32)	49 (57)	NR
Microvascular	140 (15)	12 (14)	NR
Macro and microvascular	30 (3)	25 (29)	NR
Comorbidities	86 (10)	NR	NR
Comorbidities + T2DM complication(s)	92 (10)	NR	NR
Number of T2DM complications			
None	269 (30)	NR	NR
1 T2DM complication	341 (37)	NR	NR
2 or more T2DM complications	119 (13)	NR	NR
Blood glucose level			
Random blood glucose^a^	147 (16)	NR	NR
< 200 mg/dl	73 (8)		NR
> 200 mg/dl	74 (8)		NR
Fasting blood glucose^a^	685 (76)	Mean 159 (SD 70)	NR
< 126 mg/dl	265 (30)		NR
> 126 mg/dl	420 (46)		NR
Post prandial blood glucose^a^	570 (63)	Mean 245 (SD 90)	NR
< 200 mg/dl	309 (34)		NR
> 200 mg/dl	261 (29)		NR

*NR* not reported, *NA* not available, *DQLCTQ* Diabetes Quality of Life Clinical Trial Questionnaire

^a^Reporting rate
